# Limiting collateral damage: Nosocomial infections in patients with temporary mechanical circulatory support

**DOI:** 10.1016/j.jhlto.2026.100696

**Published:** 2026-09-21

**Authors:** Ryan Dodge, Nicolas Kumar, Jonathan Tang, Amit Bardia

**Affiliations:** a Division of Cardiothoracic Anesthesiology, Department of Anesthesiology and Pain Medicine, University of Washington School of Medicine, Seattle, WA; b Mass General Brigham Department of Anesthesiology, Massachusetts General Hospital, Harvard Medical School, Boston, MA; c Department of Anesthesia, Critical Care and Pain Medicine, Ohio State University Wexner Medical Center, Columbus, OH

**Keywords:** Mechanical circulatory support, Infections, Pneumonia, ECMO, Impella, Lung transplant, Heart transplant

## Abstract

The use of temporary mechanical circulatory support (tMCS) and extracorporeal membrane oxygenation (ECMO) for patients with cardiopulmonary compromise is on the rise. While these tMCS systems provide life-saving interventions, they also pose a significant risk of nosocomial infections, which are associated with higher mortality rates. Patients receiving tMCS are critically ill and require intensive care, increasing their vulnerability to infections. Common nosocomial infections include bloodstream infections (BSI) and ventilator-associated pneumonia (VAP) in patients with ECMO and wound-related complications in patients with microaxial temporary ventricular assist devices (tVADs). Key preventative measures include the incorporation of sound ICU practices such as chlorhexidine washes, minimizing disconnections, instituting VAP bundles, and promoting enteral nutrition. The practice of continuing prophylactic antibiotics while patients are on ECMO is not routinely recommended. While treatment strategies hinge primarily on antibiotics, ensuring adequate therapeutic levels of antibiotics is challenging and may contribute to suboptimal response to systemic antibiotics in these patients. A strategy of early liberation of patients from ECMO and conversion to temporary ventricular support (temporary LVAD, RVAD, or both) may promote early extubation and patient mobilization. Whether this strategy independently reduces infectious complications remains unknown. This review examines the epidemiology and etiology of nosocomial infections in patients receiving tMCS, summarizes current prevention and treatment strategies, and discusses the impact of infection on clinical outcomes.

## Background

Patients receiving temporary mechanical circulatory support (tMCS) are a heterogeneous population due to variability in device type, indications for cardiopulmonary support, and patient comorbidities. The mechanisms and configurations of the individual devices that fall under the umbrella of tMCS vary considerably based on patients’ pathologies and treatment goals. Devices range from minimally invasive microaxial temporary ventricular assist devices (tVADs) to complex extracorporeal membrane oxygenation (ECMO) circuits with multiple centrally placed cannulae bypassing the native cardiopulmonary systems. Nosocomial infections are common in patients with tMCS devices *in situ* and result in significant morbidity and mortality in patients receiving either microaxial tVADs or ECMO.^1^, ^2^, ^3^ Beyond acute morbidity and mortality, infectious complications may prolong mechanical support, complicate candidacy for transplantation, and adversely affect subsequent clinical outcomes.^1^, ^2^, ^4^ Given the rising prevalence of tMCS devices in patients with cardiopulmonary compromise, clinicians must understand the infectious risks associated with the tMCS in the intensive care unit (ICU) as well as how infection contracted while on tMCS can impact the bridge to recovery or other destination therapies.

This review explores the epidemiology and etiology of nosocomial infections in patients receiving tMCS, discusses prevention and treatment strategies, and examines the association between infection during the peri-tMCS period and patient outcomes.

## Epidemiology

The reported incidence of nosocomial infections in patients receiving tMCS varies widely, influenced by device type, patient fragility, and site of device insertion. The existing literature focuses narrowly on either specific patient populations requiring tMCS (e.g., those listed for or following heart transplantation (HT),^5^, ^6^, ^7^ particular device types (e.g., Impella^8^, ^9^, ^10^ [Abiomed, Danvers, MA]), or singular infections (e.g., bloodstream infections (BSIs)).^5^, ^11^, ^12^ Moreover, studies utilize different definitions of nosocomial infection with additional inconsistency with respect to when tMCS was initiated and ended. The 2024 ISHLT consensus statement specifically categorizes infections in patients receiving acute MCS as MCS-specific or non-MCS-related, while commonly encountered healthcare-associated infections such as ventilator-associated pneumonia (VAP), BSI, and urinary tract infection can additionally be classified using established surveillance definitions.^13^ Because much of the existing tMCS literature predates these consensus criteria, the definitions used in individual studies should be considered when comparing infection rates across cohorts. Contemporary standardized definitions are summarized in Table 1. Outcome reporting is similarly inconsistent, with reported data varying from the proportion of infected patients within a sample population^5^, ^14^ to infection episodes per ECMO-day to capture patients that sustain multiple infections during the same tMCS course.^3^, ^11^ Illustrating this variability, a recent single-center study^4^ investigated patients bridged to HT with any type of tMCS over a 10-year period and reported a BSI rate of 15.4% (21 out of 136 patients). Another large retrospective single-center study,^15^ which included 493 patients presenting with all-cause cardiogenic shock requiring tMCS—either venoarterial (VA) ECMO initiation, Impella implantation, or both—reported a BSI incidence of 6.8%. A large multicenter study examining infection rates in patients receiving any model of Impella reported access site-related infections in 2.4% (49 out of 2,047 patients),^8^ though other types of nosocomial infection rates were not reported.

**Table 1:** Practical Definitions of Common Nosocomial Infections in Patients Receiving Temporary Mechanical Circulatory Support

Infection category	Practical definition	Relevant considerations
MCS-specific infection	Infection uniquely attributable to the MCS system, including insertion-site infection, device-related bloodstream infection, and device-associated endocarditis.^13^	Requires distinguishing device involvement from other ICU-acquired infections.
Ventilator-associated pneumonia (VAP)	Pneumonia developing after initiation of invasive mechanical ventilation without evidence of preceding infection.^32^	Particularly relevant during ECMO because prolonged ventilation, sedation, and immobility are common.
Bloodstream infection (BSI)	Laboratory-confirmed bloodstream infection not attributable to an infection at another body site.^38^	Important distinction between general BSI, central line-associated BSI, and an MCS device-related BSI.
Catheter-associated urinary tract infection (CAUTI)	Symptomatic UTI with a qualifying urine culture in a patient with an indwelling urinary catheter.^41^	Primarily reflects prolonged critical illness and ICU exposure rather than the tMCS device itself.

BSI, bloodstream infection; CAUTI, catheter-associated urinary tract infection; ECMO, extracorporeal membrane oxygenation; ICU, intensive care unit; MCS, mechanical circulatory support; tMCS, temporary mechanical circulatory support; UTI, urinary tract infection; VAP, ventilator-associated pneumonia.

Alternatively, focused investigations into infection rates among adult patients on ECMO support alone appear somewhat less heterogeneous in design but continue to have wide variability with the reporting of infections. A meta-analysis of nearly 5,000 patients reported an incidence of nosocomial infections among patients on both venovenous (VV) and VA-ECMO of 26%,^16^ with BSI and VAP being the most common etiologies. Incidences of infections reported by single-center studies ranged from 5.7% (single-center study of patients on VA-ECMO)^11^ to 64% (single-center study of patients on VA-ECMO^3^) with significant variance depending on sample size, institution, type of ECMO, and study definition of infection.^2^, ^4^, ^12^, ^17^, ^18^, ^19^ Cannula-related infections (CRIs) in patients on ECMO are also common. A single-center observational study specifically investigating CRIs—defined as signs of infection at the site of cannula insertion, positive culture taken from the site of infection or cannula tip, and infection within 30 days of cannula insertion—among patients supported on ECMO found an incidence of CRI of 17.7%.^20^

Infection rates among children on ECMO are comparable to those in adults, with recent studies reporting infection rates of 19% to 34% in pediatric ECMO populations.^21^, ^22^ Similarly, nosocomial infections are common in patients with COVID-19 needing ECMO support, with a large multicenter study reporting incidences of BSI in 44% of patients and of VAP in 69% of these patients.^18^

Infections while a patient is on tMCS are a significant concern, but this risk may persist even following tMCS removal. One study reported nosocomial infections in 140 patients out of a population of 503 patients supported by VA-ECMO (27.8%), with an additional 102 patients (20.3%) developing nosocomial infections within 28 days following removal of ECMO support.^17^ A study examining post-axillary Impella complications found that among 119 patients with an Impella 5.0 or Impella 5.5 successfully weaned and explanted, there were 6 instances of early-onset graft infections and 13 instances of late-onset graft infections (19 patients combined, 16%), with all but one requiring surgical removal of the infected graft material.^23^

Based on these data, infectious risk appears to vary according to the type and configuration of tMCS, though as stated direct comparisons between devices are limited by differences in patient populations, duration of support, and definitions of infection. ECMO appears to carry a high infectious burden, likely reflecting prolonged support, large-bore vascular cannulation, extracorporeal circuitry, frequent concomitant mechanical ventilation and invasive monitoring, and the severity of illness in patients requiring ECMO. BSI, VAP, and CRI are therefore prominent infectious complications in this population. Infections reported with microaxial tVADs more commonly involve the vascular or surgical access site. Device-specific infectious complications during IABP support appear to be less common, although contemporary infection-specific data are limited. In general, available evidence suggests differences in infectious risk and phenotype among tMCS devices but does not permit direct comparison of infection incidence across device types.

Further, the indication for tMCS and the underlying clinical context are important determinants of infectious risk and may substantially confound comparisons. Patients receiving the same support modality may have markedly different susceptibility to infection depending on the circumstances in which support is initiated (e.g., a patient supported by VA-ECMO immediately post-lung transplantation compared to a patient on VA-ECMO following complex percutaneous coronary intervention). These differences underscore that infection incidence attributed to a particular tMCS device cannot be interpreted independently of the indication for support, cannulation strategy, duration of support, surgical exposure, and host immune status. Reported infection rates should therefore be interpreted within the clinical phenotype of the population studied rather than considered an intrinsic property of the MCS device alone.

The timing of infection relative to tMCS initiation is also important when interpreting reported incidence. Infection risk is cumulative and generally increases with longer duration of device support;^24^ therefore, crude proportions of patients developing infection may not adequately account for differences in time at risk between cohorts. Early death, transplantation, recovery, or device removal may act as competing events by shortening the period during which a patient remains at risk for a device-associated infection. Conversely, infectious risk may persist following device removal, particularly when vascular graft material or other invasive devices remain in place. Reporting infection rates according to device-days and incorporating time-to-event or competing-risk analyses may therefore provide a more informative assessment of infection risk than reporting cumulative proportions alone.

As discussed above, differences in patient populations, device types, study designs, and non-standardized definitions all contribute to the wide range of reported infection rates in patients on tMCS. Despite this variability, certain distinct patterns emerge. Importantly, a significant proportion of tMCS courses are complicated by infections. The risk of infection generally increases with the duration of indwelling devices, while reported infection patterns vary among tMCS modalities. In addition, the data suggest significant infection rates following device removal. An additional limitation of the existing literature is the frequent absence of comparator groups with similar illness severity and comorbidities who are not receiving MCS. This makes it difficult to determine the extent to which reported nosocomial infections are attributable to the support device itself versus the underlying critical illness and associated invasive therapies. While data limitations preclude precise incidence estimates across all tMCS populations, nosocomial infections clearly represent a major source of morbidity and mortality, underscoring the need for standardized surveillance and infection prevention strategies in this high-risk group.

## Etiology

Discerning the category and microbial etiology of infections is of paramount importance in guiding the removal or exchange of support devices, antimicrobial treatment selection and duration, and outcome implications. Historically, standardized infection definitions in mechanical circulatory support were developed primarily for patients with durable VADs with infections categorized as VAD-specific, VAD-related, or non-VAD-related.^25^ Treatment and management pathways have been guided by these categories.^14^, ^26^, ^27^

More recently, the International Society for Heart and Lung Transplantation (ISHLT) issued a consensus statement in 2024 on definitions for both durable VADs and tMCS devices,^13^ categorizing infections among patients supported with tMCS as either MCS-specific or non-MCS-related. MCS-specific infections are defined as infections that cannot occur in patients without MCS support and include sources such as device insertion-site infections, device-related BSI, device endocarditis, and several other subcategories. Non-MCS-related infections include VAP, urinary tract infections, or other infectious etiologies not specifically related to tMCS devices. Given that this consensus statement was recently issued, limited data are available that specifically adhere to these definitions. However, much can still be interpreted from available data with regard to etiology.

A French study examining patients supported on VA-ECMO reported that 142 of 220 patients in their cohort (64%) developed infections.^3^ There were 21 cases of CRI—an MCS-specific infection—with *Escherichia coli* being the most commonly isolated organism (5 cases, 24%).^3^ The authors also reported 163 instances of VAP (most commonly isolated organism: *Pseudomonas* spp., 26%), 47 cases of BSI (most isolated organism: *Pseudomonas* spp., 21%), and 23 cases of post-sternotomy mediastinitis (most isolated organism: *Candida* spp., 35%).^3^ Another study investigating CRI in ECMO reported 39 cases among 220 patients (17.7%) with *Enterobacteriaceae* spp. being the most commonly identified organism (38%), followed by *Staphylococcus* spp. (28.2%) and *Pseudomonas* spp. (18.3%).^20^ Another study reported that 52 patients (out of 92, 55%) developed infections while supported on VA- or VV-ECMO, with CRI accounting for only 1 case (1.1%).^4^ In this same study, the most common nosocomial infection in this population was VAP (32 patients, 62%), of which the most common identified organisms were *Acinetobacter* spp. (7 cases) and *Aspergillus* spp. (6 cases).^4^ Other commonly isolated organisms include *Pseudomonas,*^12^ coagulase-negative *Staphylococcus* spp.,^12^ *Enterococcus,*^2^, ^12^ *Acinetobacter,*^17^ *Klebsiella,*^2^, ^17^ and *Candida* spp.^2^, ^3^, ^11^

Cannulae coated with bacterial biofilms may be seen in patients with bacteremia. Non-lactose fermenters such as *Flavobacterium* spp. and Carbapenem-resistant *A. baumannii* infections have been reported to be associated with biofilm formation on ECMO catheters.^28^, ^29^

To summarize, while the recently established ISHLT definitions provide a new framework for categorizing infections in tMCS-supported patients, current data remain largely retrospective and often predate the standardized classification. Nevertheless, available evidence demonstrates that non-MCS-related infections, particularly VAPs, occur more frequently than MCS-specific infections such as CRIs or device-associated endocarditis. The microbiology of these infections is highly variable, influenced by factors such as device type, duration of support, and patient comorbidities. Across available studies of patients receiving ECMO, BSIs frequently involved gram-positive organisms, particularly *Staphylococcus* and *Enterococcus*, although gram-negative pathogens were also reported.^2^, ^12^ Pulmonary infections commonly involved gram-negative organisms, including *Pseudomonas* and *Acinetobacter.*^3^, ^4^ Despite the evolving nomenclature, the clinical challenge remains the same: distinguishing MCS-specific infections from other nosocomial sources is critical for guiding antimicrobial therapy, determining the need for device exchange or removal, and ultimately improving patient outcomes. As future studies adopt these standardized definitions, a more precise understanding of infectious epidemiology in tMCS-supported patients will emerge, facilitating targeted prevention and treatment strategies.

## Preventative strategies

tMCS is rarely employed in isolation and often initiated in patients who are already critically ill. Therefore, preventing infection in tMCS patients requires a multimodal approach that not only considers the devices themselves but other common causes of infections in critically ill patients, such as VAP, central line-associated bloodstream infections (CLABSIs), and catheter-associated urinary tract infections (CAUTIs).^30^, ^31^

Prevention of VAP, CLABSIs, and CAUTIs starts with local and standardized infection control practices, with appropriate management and duration of indwelling devices being major determinants of complications.^31^ Strategies that may help reduce the risk of VAP include minimizing time on mechanical ventilation via early extubation and considering percutaneous tracheostomy as a means to decrease VAP risk when appropriate.^32^, ^33^, ^34^ Early mobilization and physical conditioning may further aid in reducing infectious complications, but require skilled multidisciplinary teams to do safely depending on the location and configuration of the tMCS circuitry.^35^ Multiple studies have shown that early mobilization even among patients with femoral cannulae in place can be performed safely in high-volume centers.^36^, ^37^ In post-transplant patients receiving tMCS, preventive strategies must also account for immunosuppression and risk of opportunistic infection, with antimicrobial prophylaxis and surveillance guided by the transplanted organ, time from transplantation, and institutional transplant protocols. Standard evidence-based CLABSI and CAUTI prevention practices should also be applied, with emphasis on daily assessment and prompt removal of unnecessary invasive devices.^38^, ^39^, ^40^, ^41^

The Extracorporeal Life Support Organization has recommended that blood, urine, and tracheal cultures in patients supported on ECMO should be obtained only when there is significant clinical suspicion of localized or systemic infection.^42^ Routine collection of these cultures, even in the later stages of ECMO support, is not supported by evidence and should be avoided in the absence of clinical signs or suspicion of infection.^42^ Repeated surveillance cultures may have difficulty differentiating between colonization and true infections, which may lead to inappropriate antibiotic utilization.^31^

Retrospective studies have not demonstrated a clear patient outcome benefit from routine antimicrobial prophylaxis following ECMO cannulation. Although peri-cannulation antimicrobial prophylaxis may be appropriate for both open and percutaneous cannulation, evidence regarding continued prophylactic antibiotics during ECMO support is mixed, and current guidelines do not recommend routine ongoing prophylaxis in the absence of another indication.^24^, ^30^, ^43^, ^44^ Peri-cannulation and ongoing antimicrobial prophylaxis practices have not been standardized across studies or ECMO centers, and differences in antimicrobial selection, timing, and duration may contribute to heterogeneity in reported infection rates.

## Treatment strategies

In the absence of positive cultures, infections in patients receiving VA-ECMO may be difficult to diagnose because conventional clinical signs, such as fever, can be attenuated or absent.^24^ A sudden worsening of shock with a concomitant marked rise in white blood cell count may be indicative of a new infection. Although infections associated with ECMO and critical illness fall along a known spectrum of organisms, empiric antibiotic selection is often tailored to local institutional practices, considering both the local prevalence of isolated pathogens and the susceptibility of the local pathogens to available antibiotics. Standardized criteria for initiation of empiric antimicrobial therapy have not been established specifically for patients receiving tMCS. When infection is clinically suspected, empiric therapy should account for the likely infectious source, prior antimicrobial exposure, duration of hospitalization and device support, local microbiology and resistance patterns, and patient-specific risk factors, with subsequent narrowing or discontinuation based on microbiologic data and clinical response. Current practices suggest a minimum of 7 days of culture-guided therapy, with consideration for extended courses in cases of slow clinical improvement or high-risk pathogens.^30^

Empiric antifungal therapy should be considered in selected patients with clinical suspicion for invasive fungal infection and relevant risk factors. Routine antifungal prophylaxis is not recommended for all patients receiving ECMO, as the incidence of invasive fungal infection in unselected adult ECMO populations is relatively low and evidence demonstrating benefit from universal prophylaxis is lacking.^30^ Antifungal prophylaxis may, however, be considered on an individualized basis in patients at particularly high risk for invasive fungal infection, including those with significant immunosuppression or other established risk factors.^42^ Patients with an open chest or prolonged postoperative critical illness may also warrant heightened clinical vigilance, although evidence supporting prophylaxis specifically based on ECMO support, chest tubes, or other thoracic drains remains limited.

Dosing of antimicrobial agents poses another challenge in these patients given the altered pharmacokinetics and pharmacodynamics in the critically ill patients in addition to possible altered volume of distribution and sequestration of drugs by ECMO circuitry. Sequestration of antimicrobials within the ECMO oxygenator may significantly impact the volume of distribution, resulting in subtherapeutic antibiotic levels.^45^ Shekar et al performed pharmacokinetic profiling of a number of commonly utilized antibiotics in patients with VA and VV ECMO and reported that failure to achieve target concentrations of these antibiotics is common.^46^ This may, at least in part, explain the lack of antibiotic response commonly seen in this patient population. It is not uncommon for tMCS patients to also require renal replacement therapy, which further complicates drug dosing. Therapeutic drug monitoring should be used if available, particularly in patients with notable hepatic or renal dysfunction, or when using drugs with very narrow therapeutic indices.^47^ Other than appropriate antimicrobials, management options beyond antimicrobial therapy are often limited. For newly diagnosed BSIs, early removal of indwelling lines is recommended to potentially restrict colonization of the MCS device. Often, this may not be possible given the limited sites available for re-siting of the invasive lines. Similarly, exchange of ECMO cannulae or the oxygenator is often not feasible.

## Prognosis and recovery

Superimposed infections in patients supported by tMCS can adversely impact patient prognosis and outcomes.^4^, ^48^ A meta-analysis of 18 observational studies examining the impact of infection during ECMO on mortality revealed a relative risk of 1.32 [95% CI 1.21, 1.44].^1^ While several studies have examined the impact of infections on ECMO patients, the distinction between VA and VV cannulation is important when evaluating ECMO-related outcomes. A retrospective single-center analysis of 206 VA- and 75 VV-ECMO patients found that patients who developed either pneumonia or bacteremia on VA-ECMO were less likely to survive the VA-ECMO run (pneumonia: 45.8% (*p* = 0.002), bacteremia: 42.9% (*p* = 0.001) vs no infection: 76.9%) or until discharge (pneumonia: 33.3% (*p* = 0.003), bacteremia: 38.5% (*p* = 0.029) vs no infection: 65.1%).^2^ In contrast, infection on VV-ECMO did not portend any increased mortality risk, though the duration of the VV-ECMO run was longer in patients who developed pneumonia or bacteremia than those who did not.^2^ Prolongation of ECMO support in the setting of infectious complications has been reported in other studies as well.^4^

There is limited literature exploring outcomes associated with infections in patients supported on microaxial tVADs. An observational study exploring the implications of microaxial VAD infection and clinical outcomes showed no difference in infection incidence among survivors and non-survivors of the Impella 2.5, CP, and 5.0 for salvage cardiogenic shock therapy.^9^

### Implications on transplant listing and outcomes

tMCS is often required as a bridge to HT for patients with end-stage heart failure. Following implementation of the revised United Network for Organ Sharing adult heart allocation policy in 2018—which assigned higher priority statuses to candidates requiring tMCS—use of tMCS devices to gain a more urgent listing status has increased.^49^ As described, nosocomial infections pose a significant risk to patients on tMCS, which can in turn impact organ listing status. A multicenter Spanish national registry of patients on tMCS (inclusive of VA-ECMO and temporary percutaneous and surgically implanted VADs) as a bridge to urgent HT found an incidence of infection of 34.9%.^6^ The patients with infections had a much greater mortality (25.3% vs 12.3%, *p* = 0.009) while on tMCS support and had a lower probability of ultimately receiving a HT (73.6% vs 85.2%, *p* = 0.025).^6^ Duration of tMCS support was the strongest variable associated with infection while on tMCS support awaiting urgent HT. As the demand for donor hearts increases, waiting times for suitable organs may lead to longer support times and increased risk of tMCS-related infections. Active infection is a contraindication for HT; thus, those patients with a newly diagnosed infection while on tMCS lose priority placement. The rationale for this restriction is that the immunosuppressive therapy required for HT can worsen infections and subsequent outcomes. Typically, patients should have the infection treated before further consideration for HT. In a separate study of HT candidates with BSI while on tMCS, all were reactivated for transplant within 72 hours of last positive blood culture and proceeded to HT in a median of 14 days from last positive blood culture.^5^ Despite the limited data on the impact of tMCS on post-HT infections, the current literature suggests that a history of cleared nosocomial infection while on tMCS does not portend greater risks for post-HT infection or mortality in those patients who ultimately undergo HT.^5^, ^6^

Conversely, for patients post-HT who require postoperative MCS support, the infectious risks associated with tMCS can have grave consequences given patients’ immunocompromised status. In a single-center retrospective study, initiation of ECMO support <24 hours after HT was significantly associated with bacterial and fungal infections within 30 and 180 days post-HT (HR = 1.7 [95% CI 1.0-2.8], *p* = 0.04).^7^ Data regarding the impact of infections on patients requiring tMCS before and after HT are limited, though evolving. Larger studies are needed to validate trends and inform best practices in the management of this cohort of patients.

The impact of infections while on tMCS as a bridge to lung transplantation has not been deeply investigated. This consideration is critical for those patients with end-stage lung disease who require both prolonged mechanical ventilation and ECMO to maintain adequate gas exchange and tissue oxygenation. A single-center retrospective analysis of 19 patients bridged to lung transplantation with ECMO included 2 patients with ECMO-related infections. Both patients died of VAP pneumonia after 22 and 15 days of VV-ECMO support.^28^ Among patients who are successfully bridged to lung transplantation with ECMO, there does not appear to be a heightened risk for post-transplant infection. A comparison of lung transplant recipients across 5 Korean centers found immediate post-transplantation infectious outcomes of ECMO-bridged recipients were similar to those of patients who were not bridged with ECMO (26.7% vs 39.4%, *p* = 0.597).^50^ However, the mortality rate of patients bridged with ECMO who developed a postoperative infection was 50%, as found by a similar study.^50^ In an evaluation of perioperative ECMO outcomes after lung transplantation, mortality was 79% (11 of 14 patients) among recipients who required postoperative reinitiation of ECMO.^50^ ECMO support was reinitiated mostly for acute respiratory failure from pneumonia, and most of these patients died from sepsis and multi-organ failure.^50^ More focused studies are needed to identify which patients are at higher risk for contracting serious infections while on ECMO and to mitigate deleterious infection-related outcomes.

## Conclusion

Infections while supported on tMCS continue to be a vexing problem, and therapeutic options are often limited. This is particularly true for patients on ECMO as infectious complications are common, difficult to treat, and have deleterious effects on patient outcomes. While there is increasing evidence highlighting this problem, prevention and treatment largely rest on sound ICU practices. Further research is needed to tailor appropriate antibiotic therapy in patients with tMCS with consideration to volume of distribution and drug sequestration issues to achieve optimal therapeutic levels. Similarly, strategies to mitigate biofilms on cannula and oxygenator surfaces are highly desirable.

Because infectious risk increases with the duration of support, early liberation from ECMO when clinically appropriate remains an important goal. In selected patients who continue to require circulatory support, transition from ECMO to temporary ventricular support may facilitate earlier extubation and mobilization and may reduce exposure to ECMO-related invasive circuitry. Whether this strategy independently reduces infectious complications remains uncertain and warrants further investigation. A multidisciplinary, guideline-based approach remains essential to mitigate infectious risks and promote best practice outcomes (Table 2).

**Table 2:** High-Yield Findings and Clinical Interpretation of Nosocomial Infections During Temporary Mechanical Circulatory Support

	High yield finding	Clinical interpretation
Overall infection burden	Nosocomial infection is common during tMCS, particularly ECMO, but reported incidence varies widely.	Crude incidence should be interpreted considering support type and duration, patient selection and comorbidities, and inconsistent definitions.
Device type	ECMO is most commonly associated with VAP, BSI, and cannula-related infection; microaxial infections are more often access or graft-related.	Infection phenotype depends partly on device configuration and invasiveness.
Clinical context	Postcardiotomy, transplant, immunosuppressed, and prolonged respiratory-failure populations have different infectious susceptibilities.	Infection risk must be interpreted within the context of indication, not attributed to the MCS device alone.
Microbiology	BSIs commonly involve gram-positive organisms; pulmonary infections more often involve gram-negative organisms; fungal infections occur in selected high-risk populations.	Empiric therapy should reflect suspected source, host factors, and local microbiology and resistance patterns.
Prevention	Evidence supports standard ICU infection-prevention practices; prolonged antimicrobial prophylaxis during ECMO has uncertain benefit.	Focus should remain on minimizing device exposure, ventilation duration, unnecessary lines, and other modifiable ICU risks.
Outcomes	Infection during tMCS is associated with worse outcomes, especially in VA-ECMO cohorts.	Infection may prolong support, delay transplantation, and increase mortality.

BSI, bloodstream infection; ECMO, extracorporeal membrane oxygenation; ICU, intensive care unit; MCS, mechanical circulatory support; tMCS, temporary mechanical circulatory support; tVAD, temporary ventricular assist device; VA-ECMO, venoarterial extracorporeal membrane oxygenation; VAP, ventilator-associated pneumonia.

## Conflicts of Interest statement

The authors declare the following financial interests/personal relationships, which may be considered as potential competing interests: Amit Bardia reports a relationship with Takeda Pharmaceuticals USA Inc that includes: consulting or advisory. If there are other authors, they declare that they have no known competing financial interests or personal relationships that could have appeared to influence the work reported in this paper.
